# Development and Pilot Test of a Multi-Component Intervention to Support Women’s Recovery from Female Genital Fistula

**DOI:** 10.1007/s00192-024-05814-3

**Published:** 2024-06-24

**Authors:** Justus K. Barageine, Hadija Nalubwama, Susan Obore, Esther Mirembe, Dianah Mubiru, Angella Jean, Susan Akori, Samuel Opio, Laura Keyser, Jessica McKinney, Abner P. Korn, Shafeesha Ali, Josaphat Byamugisha, Alison M. El Ayadi

**Affiliations:** 1 Department of Obstetrics and Gynaecology, College of Health Sciences, Makerere University, Kampala, Uganda; 2 Department of Obstetrics and Gynaecology, Kampala International University, Kampala, Uganda; 3 Department of Urogynaecology, Mulago Specialized Women and Neonatal Hospital, Kampala, Uganda; 4 Department of Social Work, Mulago Specialized Women and Neonatal Hospital, Kampala, Uganda; 5 Department of Physiotherapy, Mulago Specialized Women and Neonatal Hospital, Kampala, Uganda; 6 Department of Physiotherapy, Kawempe National Referral Hospital, Kampala, Uganda; 7 Mama, LLC, Canton, MA USA; 8 Department of Physical Therapy and Rehabilitation Science, University of California, San Francisco, CA USA; 9 Department of Obstetrics, Gynecology and Reproductive Sciences, University of California, San Francisco, CA USA; 10 School of Public Health, University of California Berkeley, Berkeley, CA USA; 11 Department of Epidemiology and Biostatistics, University of California San Francisco, 550 16th Street, Third Floor, San Francisco, CA 94158 USA

**Keywords:** Female genital fistula, Obstetric fistula, Reintegration, Rehabilitation, Holistic care approaches, Uganda

## Abstract

**Introduction and hypothesis:**

We evaluated a pilot multi-component reintegration intervention to improve women’s physical and psychosocial quality of life after genital fistula surgery.

**Methods:**

Twelve women undergoing fistula repair at Mulago Specialized Women and Neonatal Hospital (Kampala, Uganda) anticipated in a 2-week multi-component intervention including health education, psychosocial therapy, physiotherapy, and economic investment. We assessed feasibility through recruitment, retention, and adherence, acceptability through intervention satisfaction, and preliminary effectiveness through reintegration, mental health, physical health, and economic status. We collected quantitative data at enrollment, 6 weeks, 3 months, and 6 months. We conducted in-depth interviews with six participants. Quantitative data are presented descriptively, and qualitative data analyzed thematically.

**Results:**

Participants had a median age of 34.5 years (25.5–38.0), 50% were married/partnered, 42% were separated, 50$ had completed less than primary education, and 67% were unemployed. Mean number of sessions received was 12 for health education (range 5–15), 8 for counseling (range 8–9), and 6 for physiotherapy (range 4–8). Feasibility was demonstrated by study acceptance among all those eligible (100%); comfort with study measures, data collection frequency and approach; and procedural fidelity. Acceptability was high; all participants reported being very satisfied with the intervention and each of the components. Participant narratives echoed quantitative findings and contributed nuanced perspectives to understanding approach and content.

**Conclusions:**

Our results suggest that the intervention and associated research were both feasible and acceptable, and suggested certain modifications to the intervention protocol to reduce participant burden. Further research to determine the effectiveness of the intervention above and beyond surgery alone with regard to the health and well-being of women with fistulas is warranted.

**Supplementary information:**

The online version contains supplementary material available at 10.1007/s00192-024-05814-3

## Introduction

Female genital fistula is a debilitating birth injury affecting an estimated 500,000 women, mostly in sub-Saharan Africa [1, 2]. Primarily due to prolonged obstructed labor combined with a lack of high-quality emergency obstetric care access or iatrogenic etiologies, up to 100,000 new cases occur each year globally. In Uganda, 1.4% of women of reproductive age (regional range 0.5% to 4.3%) report having experienced female genital fistula symptoms in their lifetime and approximately 1,900 new cases occur per year [3].

Fistula is associated with significant physical, psychosocial, and economic consequences. Physical symptoms include uncontrollable urine and/or fecal leakage and associated odors, pain, weakness, and mobility impairments [4–7]. Fistula-causing births have high stillbirth rates [8]. Women with fistula are often stigmatized, unable to participate in social, economic, or religious activities [6, 8] and report high psychiatric morbidity [9–11]. Surgery is often transformative; however, persistent post-repair symptoms may substantially lower psychosocial health [12, 13]. These factors limit women’s ability to resume their previous roles despite successful surgery, particularly in conjunction with economic hardship [14].

Owing to the significant physical and psychological disabilities that women with fistula experience, targeted rehabilitation efforts may substantially impact recovery and quality of life. Despite the need and recognition that holistic approaches to fistula care may improve recovery, most fistula services incorporate little complementary or follow-up care [15–18]. Few studies report on the development and implementation of holistic fistula care or on the effectiveness of interventions provided as an adjunct to fistula surgical care, leaving a knowledge and practice gap [13, 19]. Studies from Nigeria, Eritrea, and Tanzania support short-term facility-based psychological intervention for improving mental health [20–23], and evidence from the Democratic Republic of the Congo and Benin supports health education and physiotherapy for improved pelvic floor strength and reducing post-repair incontinence [24–27]. Economic empowerment lacks robust evidence, yet is theoretically and anecdotally considered an important adjunct to fistula programming [13, 28] and is a primary focus of social programming outside of health care settings.

Our study sought to address the gap in evidence-based practice for reintegration following female genital fistula surgery through the development and pilot test of a multi-component intervention including health education, psychosocial counseling, physiotherapy, and economic empowerment. Intervention domains and delivery structure were informed by existing evidence and our team’s experience in surgical fistula care and research on optimizing recovery from female genital fistula surgery [29]. The objectives of this pilot study were to assess the feasibility and acceptability of the multi-component reintegration intervention in conjunction with fistula surgery in a Ugandan referral hospital and to explore its preliminary effectiveness on women’s physical and psychosocial quality of life.

## Materials and Methods

### Study Design and Site

We employed a quasi-experimental (pre/post) mixed-methods design to assess feasibility and acceptability, and to explore the preliminary effectiveness of a multi-component reintegration intervention among women accessing care for female genital fistula at Mulago Specialised Women and Neonatal Hospital in Kampala, Uganda.

### Study Participants

Eligibility criteria included being confirmed for urogenital fistula surgery, age 18 or above (or emancipated minor), and able to provide consent to study participation. Twelve study participants were enrolled in our study between October 2021 and February 2022. We invited a nested sub-sample of six study participants for in-depth interviews between December 2021 and March 2022 to understand their experience participating in the intervention and its impact. Quantitative and qualitative sample sizes were based on feasibility.

### Study Procedures

#### Participant Recruitment and Enrollment

Potential participants were identified through surgical registry review and discussion with fistula care providers. After screening for eligibility, the research team described the study goals and procedures, ensured understanding of study procedures and risks, and ascertained women’s interest in participating. Written confirmation of informed consent was obtained from all study participants.

#### Intervention

Standard fistula surgical care at Mulago Hospital includes a 2-week post-surgical hospitalization during which patients receive unstructured health education and counseling on post-repair behaviors, including instruction on pelvic floor muscle strengthening exercises. Women with significant psychiatric or physiotherapy needs are referred to specialist care.

Our pilot intervention targeted four domains to support women to overcome the physical, psychological, and economic consequences of fistula: health education, psychosocial therapy, physiotherapy, and economic empowerment (Table 1; detailed schedule in Table S1, session objectives outlined in Appendix 1). These components were selected because of their importance in the fistula literature [12, 13]. Intervention activities were planned for ~75 min per day for small group delivery (e.g., 4–8 participants). Health education sessions (*n* = 15) delivered by nurse-midwives communicated comprehensive information about the development, treatment, and rehabilitation of fistula, contraception, and birth planning for subsequent deliveries, identification of obstetric emergencies, and nutrition. Psychosocial counseling sessions (*n* = 8) delivered by a social worker sought to help women to reframe their fistula experience and prepare for returning home by developing plans for positive coping and social support development. Physiotherapy sessions delivered by physiotherapists comprised evaluation (pre-surgery, pre-discharge, and at 6 weeks following surgery) and a sequential series of facilitated exercises focused on recovering mobility, balance, and strength, and building and maintaining pelvic floor musculature (daily). Economic empowerment included a short financial literacy session by the social worker and provision of 525,000 Ugandan Shillings (equivalent to US$ 150) in investment funding.

**Table 1:** Specific components of post-surgical reintegration intervention

Component	Development, content, and rationale	Delivery structure
Health education	Development: health education activities were adapted from materials developed for health education and reproductive health counseling for women with fistula [30] and other low-literacy populations, including counseling components developed by EngenderHealth’s FistulaCare project, which were previously implemented in Eritrea [21, 30]. We have modified these materials for relevance, understandability, and acceptability to our target population as well as in consideration of participant burden for intervention participation	Facilitator: facility nurses/midwives
	Content and rationale: health education sessions focus on educational and behavioral messages designed to improve women’s understanding of fistulas and important reproductive health topics including comprehensive information about the development, treatment, and rehabilitation of fistulas, family planning and birth plans for subsequent deliveries, identification of obstetric emergencies, and nutrition (Table S1, Appendix 1). This information is intended to help women to make informed decisions during the post-repair period and beyond. Partners and/or caregivers who are helping or visiting women at the facility are included as they share the burden of fistulas with their afflicted family member are critical sources of social support, and may serve as links between the afflicted woman and other community members	Setting: group
		Sessions: during their stay at the health facility for fistula repair, each participant receives daily health education sessions: 15 sessions; no session on the day of surgery or the day after; each session lasts ~30 min; one session includes partners and/or caregivers
Psychosocial counseling	Development: short-term facility-based psychological intervention has resulted in improved mental health among women with fistulas [20, 21]. Psychosocial counseling activities build on therapeutic strategies previously developed for women with fistulas [20, 21, 31] and treatment modalities demonstrated to be efficacious for the treatment of depressive, anxiety, and traumatic stress disorders among similar populations, and that may be delivered both individually and within-group-administered therapy, including delivery by trained lay persons [32–38]	Facilitator: facility social worker
	Content and rationale: each session addresses an important component for helping women to accurately frame their fistula experience, and to prepare for returning home by developing plans for positive coping and development of social support (Table S1, Appendix 1)	Setting: group and individual sessions
		Sessions: during their stay at the health facility for fistula repair, each participant engages in 2 individual counselling sessions; 6 group counseling sessions; each session lasts ~60 min
Physiotherapy	Development: targeted physical rehabilitation can extremely reduce incontinence and increase strength [6, 24, 25]. This intervention component is based on programming developed by pelvic floor physiotherapists with expertise in the treatment of women with fistula [39]	Facilitator: facility physiotherapists supported by facility nurses/midwives
	Content and rationale: the program comprises a sequential series of exercises focused on recovering mobility, balance, and strength, and building and maintaining pelvic floor musculature. A low-literacy brochure including home exercise descriptions is provided to participants at hospital discharge to guide them through walking, range of motion, and strength-building exercises, including lumbopelvic stability and pelvic floor muscle exercises (Table S1, Appendix 1)	Setting: group and individual sessions
		Sessions: during their stay at the health facility, each participant engages in pre-surgical PT evaluation; daily physiotherapy exercises, per stage; pre-discharge PT evaluation + home treatment plan; 6-week follow-up evaluation and home treatment plan
Economic empowerment	Content and rationale: many women with fistula lack skills or investment funds to build home businesses, and many have depleted savings or incurred debt by seeking fistula care. Qualitative research suggests that small businesses, animal husbandry, or skills training might be desired [38], and supporting women in achieving their economic goals is necessary for well-being. Livelihood development programs are broadly accessible in Uganda [40, 41], and women often have prior business experience that they would like to develop further; thus, investment funding combined with a short informational session on financial literacy was seen to be the optimal strategy to meet the individual needs of women	Facilitator: facility social worker
		Setting: individual
		Sessions: at the 6-week post-surgical visit, the participant meets with the facility social worker for the following: a short informational session covering the basics of financial literacy is done on an individual basis; 525,000 Ugandan Shillings (equivalent of US$ 150) in investment funding, in cash; follow-up by the social worker to provide additional support/advice (by phone)

#### Data Collection

Mixed-methods data on intervention implementation was captured through process tracking, discussions with intervention implementers, and observation. Quantitative data were collected at enrollment (prior to fistula surgery), hospital discharge (14 days following surgery), and 6 weeks, 3 months, and 6 months following fistula repair surgery. Participants were provided with phones and monthly airtime to facilitate study communication and for remote follow-up data collection at 3 and 6 months. Qualitative data were collected between 3 and 6 months following surgery.

### Study Measures

Our primary study outcomes were feasibility and acceptability. Intervention feasibility was assessed through recruitment, retention, and fidelity/adherence. Intervention acceptability was assessed quantitatively through participant satisfaction with the intervention overall and with each intervention component using a five-point Likert-type scale. Qualitative acceptability assessment included open-ended questions on participant experiences and perspectives with the intervention overall, and with each intervention component, including perspectives on appropriateness (perspectives on fit and relevance of the innovation to the patient, problem, and setting) [42, 43]. Quantitative feasibility and mixed-methods acceptability assessment were supplemented by discussions with intervention moderators regarding challenges and adaptations required to the original intervention plan.

Preliminary effectiveness assessment was based on participant self-reported reintegration as a primary outcome [12], and secondary outcomes: physical health (incontinence [44] and level of disability [45]), mental health (i.e., depression [46], and a one-item self-esteem measure), economic stability (i.e., earnings and food security), empowerment (measured through input into household decision-making across varied domains), and stigma (enacted and internalized). Most measures were collected at each time point; however, to reduce participant burden, incontinence was assessed at baseline and at 6 weeks only, and stigma at baseline and at 6 months only. Outcome assessment was implemented at 6 months owing to our study timeframe and because this is the time point at which prior research has identified reducing gains over time [13]. Qualitative assessment included participant perspectives of intervention effectiveness.

Covariates captured and used to describe the study sample included sociodemographic characteristics (age, education, marriage/partnership status, labor force participation, income, and wealth) and duration of time with fistula), all collected at baseline.

### Data Analysis

Quantitative data on feasibility, acceptability, and preliminary effectiveness were descriptively analyzed using medians and standard deviations, medians and inter-quartile ranges, and proportions—per variable distribution. We explored trends over time using mixed-effects linear and logistic regression models, accounting for within-person clustering. Qualitative data were analyzed thematically across intervention and research components using an implementation science orientation focused on content, approach, acceptability, appropriateness, and feasibility [42]. A codebook was developed iteratively including deductive codes from our framework and interview guide and inductive codes emerging organically from the data. The codebook included a detailed description of each code, inclusion and exclusion criteria, and examples of the code in use. Transcripts were coded by three research team members a Ugandan researcher and qualitative interviewer (HN), an American mixed-methods researcher (AE) and an American Master of Public Health Student (SA). Coded data were analyzed thematically to understand participant experiences and perspectives across intervention components, and major findings and interpretation were reviewed by the co-author team. Change in economic status over time was analyzed through review of mixed-methods data.

## Results

### Study Participants

Study participants had a median age of 34.5 years (IQR 24.5–38.0; Table S2). Half had not completed primary education (6 out of 12; 50%) and half were married or in a domestic partnership (6 out of 12, 50%). Many were not employed (5 out of 12, 42%) and median monthly income was 0 (IQR 0–95,000 Ush, ~US$ 25). None had health insurance. Time with fistula varied substantially across the sample, ranging from 1 month to 23 years.

### Intervention Feasibility

The pilot reintegration intervention was considered feasible. Recruitment and retention were successful despite fewer patients seeking fistula care during the COVID-19 pandemic. Among 13 eligible individuals, 12 agreed to participate. One declined participation owing to concerns with follow-up data collection. All 12 participants were retained throughout the 6-month study.

Intervention fidelity was good, with most but not all intended sessions received. The mean number of sessions attended by intervention component was 12 for health education (range 5–15), 8 for counseling (range 8–9), and 6 for physiotherapy (range 4–8). All participants received the economic incentive. Few family members attended the final counseling session on fistula prevention and management.

Data collection, including outcome measurement, was feasible and question comprehension was good. Participants reported that the interviews were not burdensome, with only 1 suggesting shortening the quantitative survey.

Study intervention implementation was largely feasible. Participants were generally ready to engage per plan; however, a few participants preferred to postpone physiotherapy exercises for the first few days following surgery. Some counseling topics were difficult to engage with owing to their sensitive nature; however, participants reported feeling supported to discuss challenging topics. Some participants suggested that fewer sessions would be easier.

### Intervention Acceptability

Acceptability of the intervention to patients was high overall. All participants reported being very satisfied with the intervention (12 out of 12) and that each of the components were very useful (12 out of 12, for each component). Intervention moderators reported high acceptability in team discussions. Participant perspectives on the content, approach, satisfaction, appropriateness, and suggestions for each of the four components are described below, with selected quotations in Table 2.

**Table 2:** Example participant narratives on intervention content, approach, appropriateness, feasibility, satisfaction, and recommendations across components

	Health education	Counseling	Physiotherapy	Economic incentive
Content	“[The health education sessions were] helpful, especially family planning. It teaches you how to prevent another pregnancy so that you don’t get complications again.… I was taught how the fistula happened…, not to have sexual intercourse after the repair and also not to do heavy work.” P3	“She [counselor] told me that the situation changes for the better and I could move on with my life. I knew that I had hope and that the fistula will heal. So, I did what she told me; not to lose hope.” P3	“I still do those exercises up to now except today. I do them every night before sleep.” P9	“It has benefited me because I did not have a starting point but at least I got some start-up capital. I'm planning to grow my business bigger. I'm planning to set up a place where to store my things. I feel that I can get whatever I want right now. I can even plait my hair” P4
	“I learned to drink water and also not to have early sexual intercourse with a man. I also learned not to strain and also do exercises. They taught it all.” P3	“The counselling was good because she told us that we would be fine. Every time, they told me that I would be fine and that my situation would get fine. She counseled us very well.” P3	“I don’t have any problem with the physical exercises; I do them. There is a certain type which was harder [while at the hospital] and I think it is because I still had some pain, but I would still try hard. They told us to try harder because they help one to feel better. This one of raising your buttocks in the air for minutes [laughs] that one was hard. But the rest were easy.” P7	“Yes, it is useful. I prefer receiving money. I would also love to get a vocational skill-hairdressing.” P1
Approach	“I didn’t have any problem—whatever they taught, they would show you. If they talked about something, they would show you a picture.” P2	“I had no problem [in group counseling] because we gave each other time. When you are alone, they might ask a question that you don’t know the answer to. In a group, someone could be knowing the answer.” P3	“It is just one person. I would prefer to be alone. Because you could lift your legs and I don’t want people to see me.” P3	“If you observed, even people that went to school fail to get jobs. But if there is a certain type of training whereby after the training they tell you to sit somewhere and give you a job such that I’m able to earn some money, then I have no problem with that. I would love that after the training, I am sure that I am going to work somewhere and earn some money.” P2
	“If we are in group and taught about something funny, we are able to laugh together. If you are alone, you would be sad.” P2	“When the provider teaches us something nice in group, we learn and laugh together.” P2	“As we were at the ward, was how they took us. I found it nice [doing exercises in a group] because we were all patients and we would all lie down to do the exercises. I would still do them [alone] because what I wanted was to heal.” P2	“I would have liked to acquire knowledge but raising capital is the biggest problem.” P4
	“[Everyone in the group] had the same problems and no one would laugh at the other. [If I had to choose], I would prefer the group because there are questions that could be asked by another person and you wouldn’t have known about it. You find it hard when alone.” P3 (group)	“I liked [individual counseling]. It is because people might start sharing your information with anyone who cares to listen.” P2	“I appreciated it [doing exercises alone]. You see, doing these exercises sometimes involves putting your body in undesirable/indecent positions. So, much as we share the same leaking problem, I wouldn’t want my neighbor to see my extremely private parts. Therefore, I appreciated the idea of each of us doing our physical exercises privately.” P7	“[Money] has helped me a lot Eh! When I was still admitted here, I didn't have any start-up capital and yet the child insisted that he wanted to go to school. For this term, I have been able to finish his school fees. I am now going to start on this upcoming school term. If it was not for that child, my running capital would have been much bigger.” P2
	“[The mode of delivering the sessions one-on-one] is good because not everyone gets to know about your experiences. So, it is confidential. [In a group,] someone might share with other people about your problems.” P4			
Acceptability	“The health education helped us know the root cause of fistula.” P1	“I knew that I had hope and that the fistula will heal. So, I did what she told me; not to lose hope. I became strong and realized that it could happen to anyone.” P3	“I am satisfied, maybe if I didn’t do the physical exercises, probably I wouldn’t control the urine whenever I went to urinate just because I think I healed. However, they advised us to control the urine and that it is better for us. [I still do it] whenever I visit the toilet.” P7	“When I came here, I only hoped to get healed and return home. So, I was so happy getting that money because it was a surprise. Imagine getting free treatment and then receiving money after that! What kind of person would you be if you do not appreciate?” P7
	“I am satisfied because I got a lot of things from it. I also benefited from everything they told me to do. If they told you to drink or do exercises, you would heal. I learned to drink water and also not to have early sexual intercourse with a man. I also learned not to strain and also do exercises. They [health providers] taught it [health topics] all [well].” P3	“I am satisfied because you counselled me. Because of the counselling you gave me, they would tell me to do this, then I would do it.” P2	“The exercises helped me a lot because they told us that without them, there is no healing.” P1	“Giving us some start-up capital (is better)…because I did not have a job, that gave me a start-up which got me my job. By the time I came, I could not even buy myself diapers but now, I can buy them.” P4
		“She used to encourage me not to get stressed. She told me that whenever I feel stressed, I should just do physical exercises.” P4	“Yes [I’m satisfied] because they helped me strengthen my bones. Yes, I used to perform them, I would feel a little pain but it stopped with time.” P4	“I am satisfied because [the money] worked for me. If I fail to manage it properly, that won't be your (program’s) fault but mine… What was appropriate was what they (program) did; giving us some money…. I was so happy with this program.” P7
			“I found the [physiotherapy exercises] very well and in fact, I still do those exercises up to now except today. I do them every night before sleep.” P9	
Appropriateness	“I was very interested in the causes of fistula because I thought that I had been bewitched. They educated me and I was so interested in it.” P3	“She used to comfort and tell us that we could still take our drugs and stay alive. At first, I thought she was lying but when I tried everything she told me, like by not worrying, it worked.” P3	"They [exercises] helped me strengthen my bones… I used to perform them, I would feel a little pain but it stopped with time, the pain was temporary… The moment the pain stopped, I stopped doing them.” P4	“I benefited and I appreciate it because I did not expect it at all. I am satisfied with it because I wish that God helps me and it (pig) gets pregnant. I predict that in the coming months, it will be the planting season. Therefore, it is possible to get feeds from maize grain since they love maize feeds and not just grass.” P9
	“They were not easy to understand. For instance, with family planning, because I had never used it before. I was even afraid of it. I now understand it. I understood it [topics about resuming intercourse] very well. I understood it [topics about what to do in case of pregnancy] very well. What I liked most was family planning and having my delivery from a big facility… I liked everything.” P4	“I benefited because you told me that this will be done like this and that that will be done like that. Obviously, you benefit because she knows (what she is talking about).” P4	“They told us to do several exercises. Lying down and getting up every time. Those were a little hard because the wound hadn't yet healed and that is why it would bleed. It came out like this and when they covered it with a bandage, it stopped.” P2	"I would have liked to acquire knowledge but raising capital is the biggest problem." P4
			“[The exercises] weren’t hard except during the time immediately after the repair… They [physiotherapists] told us that if we did not do them, we would not heal.” P1	“It has helped me a lot Eh! When I was still admitted here, I didn't have any start-up capital.” P2
			“Some of them were hard, for example lying down to do this, it felt hard and yet the wound was still wet. Of course, we told them [physiotherapists]. They would insist, "Doing them helps to massage the wound."… We used to lie down and then they tell us "lift up, put it down… lift up, put down." So, it was harder. But now that my wound healed, they are not hard..” P2	
			“Yes [I have faced challenges when doing exercises], because when I lie down like this and try this out, I would feel like my chest is coming out, and yet when I stand upright, I quickly get tired.” P9	
Feasibility	“Honestly, in the beginning, I used to feel pain every time the provider came to teach. However, whenever I saw my colleagues going to attend, I would also used to get up although I was in pain. I would tell the provider, "let me first eat and then get up" and I would force myself up after eating to attend the session.” P2	“No [did not tell counselor that she did not clearly under the root cause of the fistula explained by social worker]. I thought that she couldn’t repeat for me.” P2	“I do them [exercises] every day before sleeping. When my children are watching TV, I just go to the bedroom and do my exercises.” P9	“The money and the physical exercises [came at the right time when I needed them the most].” P1
	“To avoid another fistula, you have to follow the instructions that they give you. You have to be patient for the number of months. The problem is resuming [intercourse] immediately. It doesn't matter even if you wait for a full year; the important thing is your life.” P2	“They [counseling sessions] were good because they were related to my health.” P4	“You could lift your legs and I don’t want people to see me. I used to tell the [physiotherapists] what I couldn’t do and they would tell me to leave it. If they had forced me then maybe I would say that.” P3	“I used some [of the money] to come back to the hospital and kept the rest because I haven’t fully recovered from the fistula. If I fully recover maybe I will use it.” P3
	It was not easy to participate in health education sessions because they could ask certain questions that I had no idea about. For example, questions about family planning” P4	“We didn’t have a single challenge with the counseling.” P9	“You see, doing these exercises sometimes involves putting your body in undesirable/indecent positions. So, much as we share the same leaking problem, I wouldn’t want my neighbor to see my extremely private parts. Therefore, I appreciated the idea of each of us doing our physical exercises privately.” P7	
	“My challenge was in the beginning when my wound was still wet. So, I felt that it was so challenging for me.… The health education can even begin 2 days after the repair.” P1		“It was not easy [doing the exercises at home]. Because in the room where I used to do them from, someone else would enter and then another person. So, it was not easy, I would perform them daily, especially at night. They would laugh at me.” P4	
			“No [I did not continue doing exercises at home]. It was [stopped] when I was still here at the ward. It is because they did not give me the document [for the exercises]. Yes, [if I had got the document] I would have performed them [exercises].” P1	

#### Health Education

Study participants generally reported understanding the health education content well and recalled key content areas such as how a fistula occurs and primary discharge instructions (e.g., delaying post-repair sexual intercourse, avoiding heavy work, drinking enough water, eating healthily, and doing exercises). Participants also reported being counseled on postponing post-repair pregnancy and the importance of cesarean section for subsequent births. One participant reported some difficulty with understanding the cause of their fistula, and 2 participants particularly appreciated the family planning information given their lack of prior knowledge. Participants reported that the educational content was very useful for them, met their needs, and was understandable, and thus was considered appropriate. Participants generally appreciated the approaches used within the health education sessions. One participant specifically mentioned enjoying the handouts shared by the moderators. Most participants preferred group versus individual sessions owing to the social support provided by individuals with the same problem; however, one participant who had participated individually voiced potential confidentiality concerns with a group format.

#### Counseling

Participants felt that counseling covered important topics for overcoming fistula-related challenges. The counseling content was useful for them, motivated them, and met their needs (appropriateness). Some participants preferred the group counseling approach whereas others preferred individual counseling. Participants who preferred group counseling believed that the group facilitated greater learning. Participants who preferred individual counseling prioritized confidentiality. Overall, participants were satisfied with counseling. They expressed being comforted during counseling and trusted the counselor.

#### Physiotherapy

Participants appreciated the physiotherapy content, including exercise training. The physiotherapy was considered helpful and appropriate for fistula patients, and participants appreciated physical improvements after initiating physiotherapy, with some respondents also noting stress relief. Some participants, mainly those who underwent abdominal surgery, reported that certain exercises were painful, particularly early after surgery. Some suggested that physiotherapy exercises might be delayed by a few days or weeks to minimize the pain and allow the surgical wound to heal.

Preferred physiotherapy session mode (approach) varied, with some participants preferring individual sessions and others preferring group sessions. Some respondents feared being made fun of doing exercises, exposing their private parts, or failing to do some exercises. Others preferred group sessions for social support and encouragement.

All respondents expressed satisfaction with the exercises mainly because they believed that they helped them heal faster. However, certain exercises were more painful or difficult. Many respondents reported continuing the physiotherapy exercises beyond their hospital stay for fistula repair in order to heal. Some participants failed to continue with the exercises at home, simply because they forgot the exercises that they were meant to do, and a few missed getting discharge cards describing the different exercises.

#### Economic Incentive

All participants appreciated and were satisfied with the economic incentive they received as a start-up fund, and most did not expect it. Most started small businesses and reported that the amount given to them ($150) was adequate. Some participants suggested adding vocational skills training (e.g., hairdressing or baking). Most preferred money versus vocational skills training as it did not guarantee employment, whereas money met immediate family needs. Others suggested adding follow-up via phone or visits to check on business progress and well-being.

The small businesses included roadside food stalls, general trading, animal rearing or farming, and crafts. They focused on a range of commodities including charcoal, rice, and groundnuts, coffee beans, pigs, silverfish, chicken, and clothing. One participant reported investing in a popcorn machine and now makes and sells popcorn. Another participant brewed and sold alcohol. Two participants described using the funds to support other family members or household expenses such as school fees, rent, clinical care, or family funeral expenses. One participant stayed with family post-surgically and saved the money to start a restaurant after she returned home.

#### Research

Participants found the research approach acceptable. Some participants perceived the survey as short, whereas others noted that it was long; however, participants reported no difficulty with the questionnaire and interview length or returning to the health facility for follow-up data collection. They appreciated facilitation of their return transportation for data collection.

### Preliminary Effectiveness

Reintegration score was mean 34.1 (SD 8.1) at baseline and increased to 65.4 (SD 6.9) at 6 months post-surgery. This increase represented a statistically significant trend over time, with the mean monthly increase of 4.4 points (95% CI 3.01–5.90). Secondary outcomes also suggested improving trends. For example, the mean disability score was 15.7 (6.0) at baseline and reduced to 1.8 (2.2) at 6 months post-surgery (mean 2.1-point decrease each month, 95% CI −2.79, −1.36). Mean depressive symptoms were 12.9 (SD 5.0) at baseline and reduced to 2.4 (SD 3.2) at 6 months post-surgery (mean 1.4-point per month reduction, 95% CI −2.04, −0.79). Mean self-esteem increased from 2.3 (SD 1.1) at baseline to 4.5 (SD 0.9) at 6 months, reflecting significant improvement.

At baseline, all participants were leaking urine (*n* = 12, 100%); at 6 weeks post-surgery, only 1 person (8.3%) reported ongoing urine leakage (not shown). Median impact of urine leakage interference on everyday life reduced from the scale maximum of 10 (IQR 9–10) at baseline to the scale minimum of 0 (IQR 0–0) at 6 weeks. Respondent input into household decision making exhibited little change over time, except for food crop and livestock farming (80% to 86% from baseline to 6-month follow-up, *p* = 0.006) and nonfarming economic activities (75% to 100%, marginal *p* = 0.091).

We observed important reductions in stigma experiences and increases in social support. From baseline to 6 months, enacted stigma reporting decreased from 6 to 2, reporting being treated differently than before fistula, 5 to 1 reporting being treated badly, and 6 to 1 reporting abandonment owing to the fistula. Internalized stigma reduced from 9 at baseline to 2 at follow-up, and similar numbers reported choosing to avoid others or join social activities owing to the fistula (6 to 1). Over the study follow-up, consistency in emotional support increased from 6 to 10 and informational support increased from 4 to 7, yet tangible support decreased from 6 to 5 (Table 3).

**Table 3:** Change in primary and secondary outcomes across pilot study participants, and trends over time

	Mean (SD)^a^, median (IQR) or proportion by time since surgery				Trend over time		
	Baseline	6 weeks	3 months	6 months	β^a^/OR^b^	95% CI	*p* value
Primary outcome							
Reintegration score^**a**^	34.1 (8.1)	60.3 (6.0)	64.4 (10.0)	65.4 (6.9)	4.45	(3.01, 5.90)	< 0.001
Secondary outcomes							
Incontinence impact	10.0 (9.0–10.0)	0.0 (0.0–0.0)	–	–	–	–	< 0.001
Disability^a^	15.7 (6.0)	5.3 (4.8)	–	1.8 (2.2)	−2.08	(−2.79, −1.36)	< 0.001
Depressive symptoms^a^	12.9 (5.0)	2.3 (2.6)	1.3 (1.8)	2.4 (3.2)	−1.42	(−2.04, −0.79)	< 0.001
Self-esteem^a^	2.3 (1.1)	4.6 (0.5)	4.4 (1.2)	4.5 (0.9)	0.29	(0.14, 0.45)	< 0.001
Food security^a^	14.5 (9.0)	8.6 (6.4)	9.8 (7.9)	6.2 (7.3)	−1.18	(−2.02, −0.34)	0.006
Input into household decision making							
Food crop and livestock farming^b,^ ^c^	4 (80%)	4 (80%)	5 (63%)	6 (86%)	11.15	(1.99, 62.61)	0.006
Cash crop farming^b, d^	1 (50%)	1 (100%)	1 (33%)	2 (100%)	–	–	–
Nonfarming economic activities^b,^ ^e^	6 (75%)	8 (73%)	8 (89%)	11 (100%)	1.96	(0.90, 4.28)	0.091
Wage and salary employment^b,^ ^f^	5 (83%)	4 (67%)	1 (33%)	1 (100%)	4.72	(0.44, 50.29)	0.198
Major household purchases ^b,^ ^g^	7 (70%)	6 (50%)	7 (58%)	9 (75%)	1.28	(0.81, 2.04)	0.297
How own income will be used^b,^ ^h^	7 (58%)	9 (75%)	7 (58%)	7 (58%)	0.94	(0.69, 1.29)	0.720
Health care for self^b,^ ^i^	7 (58%)	9 (75%)	7 (58%)	7 (58%)	0.94	(0.69, 1.29)	0.720
Visits to family or relatives^b,^ ^j^	7 (58%)	9 (75%)	7 (58%)	7 (58%)	0.94	(0.69, 1.29)	0.720
Stigma							
Enacted^k^	1.0 (1.0–3.0)	–	–	1.0 (1.0–1.0)			0.037
Internalized^k^	4.0 (1.5–4.0)	–	–	1.0 (1.0–1.0)			0.001

^a^β from linear regression and ^b^odds ratio from logistic regression

Scale ranges were reintegration 0–100, disability 12–60, depressive symptoms 0–27, self-esteem 1–5, incontinence impact (0–10), enacted stigma (1–4), and internalized stigma (1–4)

Different sample sizes (*n*) across involvement in some household decision-making: ^c^baseline: 5, 6 weeks: 5, 3 months: 8, 6 months: 7; ^d^baseline: 2, 6 weeks: 1, 3 months: 3, 6 months: 2; ^e^baseline: 8, 6 weeks: 11, 3 months: 9, 6 months: 11;^f^baseline: 6, 6 weeks: 6, 3 months: 3, 6 months: 1; ^g^baseline: 10, 6 weeks: 12, 3 months: 12, 6 months: 12; ^h^baseline: 12, 6 weeks: 12, 3 months: 12, 6 months: 12; ^i^baseline:, 6 weeks: 12, 3 months: 12, 6 months: 12; ^j^baseline: 12, 6 weeks: 12, 3 months: 12, 6 months: 12

Definitions of different categories: food crop: crops or livestock grown primarily for house or for household food consumption; cash crop: crops grown primarily for sale in the market; nonfarming economic activities: small business, self-employment, buy and sell; wage and salary: in kind of monetary work, both agricultural and other wages

Changes in living status, relationship status, individual financial status, and household financial status over the study period are described in Table 4. Change in living status was reported by most participants at 6 weeks (8 out of 12) and 3 months (8 out of 12) post-surgery, most of which was positive and focused on stigma reduction; fewer participants endorsed changes in relationship status, yet comments in this domain were similarly focused on stigma reduction. Most participants reported positive changes in individual employment, income, savings, and debt at 3 (8 out of 12) and 6 months (10 out of 12), and positive household changes reported largely reflected changes in individual income.

**Table 4:** Changes reported in living status, relationship status, individual financial status, and household financial status over time

	6 weeks	3 months	6 months	Notes on change
Living status	Participants reporting change felt free to move without worrying about urinary incontinence. They live in clean environments and sleep well (8 participants)	Most participants reported continued positive change including feeling better, interacting with others, and working. One individual reported being treated poorly by her mother (who she lives with; 8 participants)	Participants experienced ongoing positive changes, including getting what they need and interacting well with people. One participant’s husband left her (4 participants)	Change over time followed different patterns. Change at only one point was reported by 4 (6 weeks only), 3 (3 months only), and 1 (6 months only). 2 reported change at 6 weeks and 3 months but not at 6 months, and 2 reported change at all time points
Relationship status	Participant does not feel rejected by community (1 participant)	Participants report reductions in stigma and improved community relationships (4 participants)	One participant reiterated free conversation with peers as positive whereas another separated from her husband (2 participants)	The participant noting change at 6 weeks also reported change at 3 and 6 months. Two participants reported change at both 3 months and 6 months, whereas 2 others reported change only at 3 months
Individual employment, income, savings and debt	Participants report resuming economic activity including selling vegetables and rearing chickens, which allows for profit or savings (2 participants)	Participants continue to return to engagement in economic activity, including food stalls, crafts, bars, animal rearing, etc. This has resulted in increased earnings and the ability to save (8 participants)	Participants continued to expand economic activities, working, and saving. Some negative impacts reported include increased financial responsibility due to husband losing work, another who had to cover family funeral expenses, reduced sales due to seasonal changes in community disposable income (school fees), and a fourth whose husband stole her study money and took over her business (10 participants)	5 participants reported change at 3 months and 6 months only; 3 reported change at 6 months only; 1 reported no change; 2 reported change at all time points, whereas 1 reported change at 3 months only
Household income, savings, and debt	One participant reiterated individual income changes whereas another shared that she was able to save (2 participants)	Participants contribute to household income and savings. One participant’s crochet business improved their household income and savings. Another participant used their income to clear a relative’s debt. One participant also expressed that that they now equally contribute to running their household financially along with their husband (7 participants)	Some participants continue to generate income and increase savings through small businesses. Other participants’ average household income decreased because their husband lost their job or live alone and are unemployed (6 participants)	No change was reported by 4 participants. 2 reported change at all time points; 1 reported change at 3 months only; 1 reported change at 6 months only; 1 reported change at 6 weeks and 3 months only, whereas 2 reported change at 3 and 6 months only

Within 3 months of receiving the incentive, participants created small businesses (see acceptability), saved the money, used it for household essentials, or paid debt and taxes. By 6 months, many participants were involved in income-generating activities. Assets did not change over time but investments improved household income and savings. Some participants experienced an improvement in the quality of their living environment. They expressed better living conditions such as sleeping well in clean beds, working, and buying what they needed.

## Discussion

Establishing feasibility and acceptability of an intervention and the surrounding research are key foundational components required before moving forward with robust assessment of intervention effectiveness. We found that a multi-component fistula reintegration intervention incorporating health education, psychosocial counseling, physiotherapy, and an economic incentive delivered within a public Ugandan referral hospital setting at the time of fistula surgery was demonstrated to be feasible and acceptable to both implementers and recipients. Furthermore, the research structure around the intervention for assessing effectiveness was also considered feasible and acceptable to implementers and participants. Research findings informed important adjustments to the original intervention protocol to reduce participant burden and improve engagement. Our research contributes to the limited evidence based on holistic fistula care, which supports the need for reintegration programming addressing the unique physical, psychosocial, and economic needs of women recovering from a fistula and fistula surgery [2].

Implementing fistula care has its own unique challenges, which are structured by the relatively low incidence but broad geographic distribution of patients, the constellation of physical and social sequelae experienced by individuals with the condition, in conjunction with traditionally siloed care models for these needs, the low socioeconomic status and health literacy of this population, and the lower-resource settings in which a fistula more commonly occurs. These challenges, relevant to both fistula surgery and reintegration programming, were considered by our research team and have similarly influenced the scope of other reintegration interventions reported in the literature. Other programs providing reintegration support have tested health education, psychosocial counseling, physical rehabilitation, social immersion, and livelihood improvement, alone or paired—and largely at the fistula repair facility [13, 47, 48].

We sought to be comprehensive in our reintegration programming approach through addressing physical and psychosocial health and economic status, employing group delivery strategies to capitalize on shared experiences and build social support, extending certain sessions to family members to strengthen community-based support, and providing an economic incentive for women to use as they desired; however, certain limitations to our design should be explored in future research and implementation. Optimal reintegration programming should extend facility-based services to community settings given the important role of the unique community environments of each individual to accommodate their individual needs, and facilitators and barriers to reintegration, which include social support networks and resources. We implemented our intervention within the fistula surgical facility to exploit the 2-week facility-based recovery period and because of service provision challenges across large distances. Building community networks and programming within this model was not possible within the scope of our small grant mechanism, but a systems approach should be prioritized for country-level reintegration program developers and implementers. Broad engagement of community health workers has been feasible within the Fistula Foundation’s Fistula Treatment Network in Kenya for surgical mobilization and follow-up [47], and other models for expanding care continuity through existing community-based services are needed. Finally, most existing reintegration programming supporting economic stability offers vocational training, with or without added financial support; owing to the referral-facility base of our intervention and geographic diversity of participant residence, we chose not to coordinate further vocational programming. This design increased implementation feasibility within our clinical setting, but further consideration should be given to connecting women with existing vocational training programs as needed to improve economic status.

Our preliminary effectiveness results identified improving trends across time within key domains of interest; however, our pilot study was not designed to test the effectiveness of the reintegration intervention components separately from the impact of fistula surgery, which we know has significant impact on improving women’s physical and psychosocial health [49, 50]. The findings of the current research confirm that the research structure around our intervention is both appropriate and feasible, but cannot be considered evidence of intervention effectiveness.

Key limitations to this study include our implementation within one site, small sample size, and lack of a comparison group; although appropriate for a pilot study such as ours, these factors did not allow us to explore feasibility and acceptability differences across diverse settings and populations and limited our ability to formally assess intervention effectiveness. Pilot study implementation and data collection occurred during the COVID-19 pandemic. Local mitigation measures included travel restrictions, reduced medical capacity, and limitations on elective surgery provision, which influenced our ability to implement this study as planned. Finally, although our study evaluated outcomes at 6 months, which is the time point at which prior longitudinal research has identified reducing physical and psychosocial gains, a longer evaluation period may be more appropriate to fully capture this impact, as well as any economic impact [13].

## Conclusion

Holistic approaches to genital fistula programming are important for supporting women to overcome the significant physical and psychological disabilities associated with this condition in conjunction with surgery, yet the evidence base is limited. Our facility-based multi-component intervention, built on prior literature, theory, and field experience, was found to be both feasible and acceptable within pilot research. Further research to determine the effectiveness of this intervention above and beyond surgery on the health and well-being of women with fistulas is warranted. Future research should employ study designs capable of evaluating the value added of reintegration components on key fistula outcomes. Quality of research design and reporting has been limited for many fistula interventions [2, 13], and integrating robust research structures with ongoing and new reintegration programming is an important strategy for improving the quality of this evidence base so that others can learn from this body of literature, including attention to implementation considerations such as human resources and costs, to meet the needs of policy makers and program leadership, and ultimately improve women’s recovery from fistula.

### Electronic supplementary material

Below is the link to the electronic supplementary material.

Supplementary file1 (DOCX 62 KB)

## Data Availability

De-identified data are available from the corresponding author on request.

## Appendix 1. Reintegration intervention session details for health education, psychosocial counseling, and physiotherapy components

### Health education

**Table:**

Session	Objective	Duration	Timing^a^	Delivery
1. Admission and pre-operative management: preparing for upcoming surgery and recovery (part 1)	Introduce patients to fistula	30 min	Pre-operatively	Group
	Introduce patients to care providers			
	Provide overview of procedure, risks, and outcomes			
	Address patient concerns and questions about procedure and recovery		Day 1	
2. Admission and pre-operative management: preparing for upcoming surgery and recovery (part 2)	Familiarize patients with the patient ward and the surgical theater	30 min	Pre-operatively	Group
	Discuss pre-operative preparations			
	Discuss post-operative management			
	Address any patient concerns and questions about procedure and recovery		Day 2	
3. Overview of post-operative management	To discuss outcomes of surgery	30 min	Day 2 (post-operatively)	Group
	To discuss post-operative care management at the facility			
	Address any patient concerns and questions			
4. Reproductive system anatomy and function	Familiarize participants with the female reproductive system and female pelvis, anatomy, and functions	30 min	Day 2 (post-operatively)	Group
5. Development of fistula	Identify the causes of obstetric fistula	30 min	Day 4 (post-operatively)	Group
6. Common myths and misconceptions about fistula	To dispel any common rumors and myths about obstetric fistula	30 min	Day 4 (post-operatively)	Group
	To explain how these may hamper women’s ability to prevent fistulas and to access treatment			
7. Health and social consequences of fistula	To identify and discuss the health consequences of fistula	20 min	Day 6 (post-operatively)	Group
	To identify and discuss the social consequences of fistula			
8. Fistula prevention	To discuss ways of preventing obstetric fistula after repair	40 min	Day 6 (post-operatively)	Group
	To introduce birth spacing			
9. Sexual and reproductive health after fistula repair	To educate patients on sexual health	30 min	Day 8 (post-operatively)	Group
	To familiarize participants reproductive tract issues			
	To identify personal timelines for family planning and fertility preferences			
10. Family planning	To identify different family planning methods	30 min	Day 8 (post-operatively)	Group
	To dispel myths and misconceptions regarding contraceptives			
	To facilitate discussion of which family planning method might be right for the participant			
11. Future obstetric care	To understand the components and reasons for antenatal care during pregnancy	30 min	Day 10 (post-operatively)	Group
	To recognize the danger signs during pregnancy			
	To discuss the importance of planning and preparation for safe delivery			
	Facilitate participant creation of a birth plan			
12. Nutrition	To understand optimizing nutrition for recovery	30 min	Day 10 (post-operatively)	Group
13. Post-discharge management	To identify ways in which to take care of herself at home	30 min	Day 12 (post-operatively)	Group
	To discuss the importance of follow-up visits			
	To recognize surgical warning after discharge			
	Facilitate participants in creating a complication			
14. Recap of health education sessions	To prepare the patient for her return home, which maximizes the likelihood of a positive reintegration experience	30 min	Day 12, 13, or 14 (post-operatively)	Group
	To review all of the materials and information discussed during the health education sessions			
	For participants to share their experiences, ask questions			
15. Health education counseling for the participant’s family/caregiver (optional)	To communicate patient’s health condition and treatment (only if requested to be shared with family member(s)/caretaker	60 min	TBD	Group
	To communicate any risks and possible side effects			
	To address any concerns and questions brought up by the patient’s social support			
	To identify warning signs			
	To develop a plan in the case of complications			
	To provide guidance on how to support the patient at home			
	To discuss sexuality after surgical repair			
	To discuss reproductive tract and sexually transmitted infections			

*TBD* to be decided

### Psychosocial counseling

**Table:**

Session	Objective	Duration	Timing^**a**^	Delivery
1. Introduction to psychological counseling	Introduction to counseling	30 min	Day −1 (pre-operatively)	Individual
	Assessing the patient's needs and concerns			
	Set initial treatment plan/goals			
	Determine if the patient is ready for group counseling session			
2. Recounting experience with fistula	To get familiar with each other	60 min	Day 2 (post-operatively)	Group
	To normalize the patient’s experience			
	To acknowledge the negative and possible positive impacts of the fistula experience on relationships with others			
	To begin exploration of how the patient’s experience of an obstetric fistula impacts her feelings and thoughts about herself			
3. Reframing the fistula experience	To promote cognitive reframing of the fistula experience through medical education	60 min	Day 4 (post-operatively)	Group
	Teach medical information on fistula and surgery			
	Validate experiences of a normal medical condition			
	Help the patient to reframe her personal narrative			
4. Identifying thoughts and emotions	To introduce the patient to the cognitive-behavioral model	60 min	Day 6 (post-operatively)	Group
	To begin the teaching patient how to reframe negative or unhelpful thoughts			
	Review key emotions			
	Identify negative and problematic thoughts			
	Link thoughts to resulting emotions			
	Begin practicing reframing problematic thoughts			
	To manage the emotions associated with their situation			
5. Develop coping strategies	To help the patient to recognize and respond to stressors by utilizing appropriate and effective coping skills	60 min	Day 8 (post-operatively)	Group
	Discuss the patient’s negative vs positive coping strategies			
	Coach the patient to recognize stressors and respond by utilizing appropriate and effective coping skills			
6. Explore social relationships	To examine the effect of social relationships on the patient’s life	60 min	Day 10 (post-operatively)	Group
	To generate specific strategies to strengthen social relationships			
	Discuss how one’s social network influences thoughts and feelings			
	Formulate strategies to expand and optimize the patient’s social support network			
	Review tools for discussing her condition with others			
	To practice communication skills			
	Mobilize home support by identifying partner/caregiver to participate in optional combined counseling session			
7. Planning for the future	To prepare the patient for her return home, which maximizes the likelihood of a positive reintegration experience	60 min	Day 12 (post-operatively)	Group
	Make practical plans for the patient’s return to her community			
	Discuss the possibility of an incomplete cure and post-surgery medical recommendations			
8. Patient pre-discharge assessment	Review therapy action plan and discuss progress in reaching goals	30 min	Day 14 (post-operatively)	Individual
	Patient assessment and addressing the patient's remaining needs and concerns			
	Identify if the patient requires additional psychiatric counseling/emotional support/medication if she suffers from a trauma-related fistula			
9. Building social support opportunities (optional)	Intended to facilitate social support for the patient returning home after repair	60 min	Offered between days 10 and 15 (post-operatively)	Group (with patient and partner/caregiver)

### Physiotherapy

**Table:**

Session	Objective	Duration	Timing	Delivery
1. Introduction to physical therapy	Introduction to physical therapy	60 min	Day −2 (pre-operatively)	Individual
	Introduction to functional pelvic anatomy			
	Goal setting and expectations			
	Introduce breathing exercises			
	Complete baseline WHODAS and ICIQ with physical therapist			
2. Physical therapy assessments	Functional mobility assessment	60 min	Day −1 (pre-operatively)	Individual
	Practical abdominal assessment			
	External pelvic floor assessment			
	Internal pelvic floor assessment			
3–8. Physical therapy for acute/inflammatory phase	Functional mobility exercises	60 min	Days 2–7 (post-operatively)	Group
	Diaphragmatic breathing exercises to load the pelvic floor without straining the healing tissues			
	Sub-maximal pelvic floor muscle exercises while lying in bed			
9–14. Physical therapy for sub-acute phase	Functional mobility exercises	60 min	Days 8–13 (post-operatively)	Group
	Diaphragmatic breathing exercises			
	Sub-maximal to maximal pelvic floor muscle exercises			
	Exercises for the abdominal, hip, and low back muscles			
	Stretching areas of scar tissue and muscle and joint tightness (or contracture)			
15. Physical therapy for sub-acute phase with treatment plan for discharge	Group: same objectives as sessions 9–14	60 min	Day 14 (post-operatively)	Group and individual
	Individual: patient assessment and development of home treatment plan			
Follow-up	Physical assessment	60 min	6 weeks	Individual
	Reinforcement/adjustment of exercises			

*WHODAS* World Health Organization Disability Assessment Schedule, *ICIQ* International Consultation of Incontinence Questionnaire
