# Supplementary material for: Development and Pilot Test of a Multi-Component Intervention to Support Women’s Recovery from Female Genital Fistula
Source: Int Urogynecol J. 2024 Jun 24;35(7):1527–47. doi: 10.1007/s00192-024-05814-3 (PMC11315714; doi:10.1007/s00192-024-05814-3)
Supplement: Supplementary file 1 — Supplementary file1 (DOCX 62 KB) [file 192_2024_5814_MOESM1_ESM.docx]

**SUPPLEMENTARY TABLES**

**Table S1. Planned Intervention Schedule**

| **Component** | **Pre-op 1** | **Pre-op 2** | **Surgery** | **Post-op 2** | **Post-op 3** | **Post-op 4** | **Post-op 5** | **Post-op 6** | **Post-op 7** |
| --- | --- | --- | --- | --- | --- | --- | --- | --- | --- |
| **Session No.** | **1** | **2** | **& Post-Op 1** | **3** | **4** | **5** | **6** | **7** | **8** |
| HE | Admission and pre-op mgmt, 30m | Pre-op mgmt, 25m | Surgery + Post-op Day 1: No intervention sessions | Post-op mgmt, 30m  Repro system anatomy & function, 30m |  | Fistula causes, 30m  Common fistula myths and misconcept, 30m |  | Health and social consequences of fistula, 20m  Fistula prevention, 40m |  |
| PC | Intro to counseling, 40m *individual* | |  |  | Explore fistula experience, 60m |  | Cognitive reframing of fistula experience, 30m |  | Identifying thoughts and emotions, 40m Reframing problematic thoughts, 20m |
| PT | Intro to PT, 20m  Mobility and pelvic floor assessment, 15m Daily: Breathing & functional mobility exercises, 15m | |  | Daily: Breathing & functional mobility exercises, 15m | | | | | |
| Total time | 75m + 40m ind | 75m + 40m ind | 0m | 75m | 75m | 75m | 75m | 75m | 75m |

| **Component** | **Post-op 8** | **Post-op 9** | **Post-op 10** | **Post-op 11** | **Post-op 12** | **Post-op 13** | **Post-op 14** | **Other Sessions** |
| --- | --- | --- | --- | --- | --- | --- | --- | --- |
| **Session No.** | **9** | **10** | **11** | **12** | **13** | **14** | **15** | **TBD** |
| HE | Sexual/RH after fistula, 30m  Family planning, 30m |  | Obstetric care, 30m  Nutrition, 30m |  | Post-discharge management, 30m  Recap, 30m | Recap, 30m | Recap, 30m | Health education for family/caretaker, 60m |
| PC |  | Coping skills, 40m  Social support and relationships, 20m |  | Planning for future, 60m |  | Pre-discharge review, 60m *individual* | | Building social support with family/caretaker, 60m |
| PT | Daily: Breathing & functional mobility exercises, 15m | | | | Daily: Breathing & functional mobility exercises, 10m Individual assessment, treatment plan, 40m | | Daily: Breathing & functional mobility exercises, 15m | Individual assessment, treatment plan, 40m @ 6w |
| Total time | 75m | 75m | 75m | 75m | 75m + 40m individual | 45m + 40m ind | 45m + 40m ind |  |

Notes: *EE: Economic empowerment component distributed at the 6-week post-repair visit.* *HE: Health Education, PC: Psychosocial Counseling; PT: Physiotherapy; EE: Economic Empowerment*

**Table S1. Participant Sociodemographic Characteristics**

|  | N=12 |
| --- | --- |
| **Age** |  |
| Median (IQR) | 34.5 (24.5-38.0) |
| Min, Max | 19, 42 |
| **Education** |  |
| None | 1 (8%) |
| Some primary | 5 (42%) |
| Completed primary | 3 (25%) |
| Some secondary | 1 (8%) |
| Vocational | 2 (17%) |
| **Relationship status** |  |
| Married or domestic partnership | 6 (50%) |
| Widowed | 1 (8%) |
| Separated | 5 (42%) |
| **Employment** |  |
| Informal employment | 1 (8.3%) |
| Self employed | 3 (25.0%) |
| Housewife | 3 (25.0%) |
| Not employed | 5 (41.7%) |
| **Average monthly income from formal or informal business activities** |  |
| Median (IQR) | 0 (0-95,000)^b^ |
| Min, Max | 0, 350,000^c^ |
| **Individual Assets** |  |
| Have a bank account | 2 (17%) |
| Own a mobile phone | 7 (58%) |
| Own land | 2 (17%) |
| Own animals | 1 (8%) |
| Own a business | 3 (25%) |
| Participate in savings/investment groups | 2 (17%) |
| Hold health insurance | 0 (0%) |
| **Length of time with fistula** |  |
| <3m | 4 (33.3) |
| 3-12m | 3 (25.0) |
| 1-3y | 3 (25.0) |
| More than 3y^a^ | 2 (16.6) |

*Notes: ^a^9 and 23 years, respectively. ^b^95,000 Ush equivalent to $24 USD; ^c^350,000 Ush equivalent to $95 USD.*
